# Mechanosensation induces persistent bacterial growth during bacteriophage predation

**DOI:** 10.1128/mbio.02766-22

**Published:** 2023-11-01

**Authors:** Guy Mason, Matthew J. Footer, Enrique R. Rojas

**Affiliations:** 1 Department of Biology, Center for Genomics and Systems Biology, New York University, New York, New York, USA; 2 Department of Biology, Howard Hughes Medical Institute, University of Washington, Seattle, Washington, USA; University of Maryland, College Park, Maryland, USA

**Keywords:** cell mechanics, microbial ecology, bacteriophage T7, microfluidics, Rcs pathway

## Abstract

Although the relationship between bacteria and lytic bacteriophage is fundamentally antagonistic, these microbes not only coexist but thrive side by side in myriad ecological environments. The mechanisms by which coexistence is achieved, however, are not fully understood. By examining *Escherichia coli* and bacteriophage T7 population dynamics at the single-cell and single-virion level using a novel microfluidics assay, we observed bacteria growing “persistently” when perfused with high-titer bacteriophage. Bacteriophage persistence occurred at a frequency five orders of magnitude higher than is expected from the natural selection of bacteriophage-resistant mutants. Rather, the frequency of persistence was correlated with the degree to which the bacteria were mechanically compressed by the microfluidic perfusion chamber. Using a combination of mutagenesis and fluorescent imaging techniques, we discovered that compression induces persistence by activating the Rcs phosphorelay pathway, which results in the synthesis of extracellular capsule that sterically blocks bacteriophage adsorption. Other forms of mechanical perturbation also promoted Rcs activity and persistence. These findings have important implications for our understanding of microbial ecology in many important environments, including the gut and the soil, where bacteria grow in confinement.

**IMPORTANCE:**

Bacteria and bacteriophage form one of the most important predator-prey relationships on earth, yet how the long-term stability of this ecological interaction is achieved is unclear. Here, we demonstrate that *Escherichia coli* can rapidly grow during bacteriophage predation if they are doing so in spatially confined environments. This discovery revises our understanding of bacteria-bacteriophage population dynamics in many real-world environments where bacteria grow in confinement, such as the gut and the soil. Additionally, this result has clear implications for the potential of bacteriophage therapy and the role of mechanosensation during bacterial pathogenesis.

## INTRODUCTION

Lytic bacteriophages shape bacterial populations in many important environments, including the gut (1), the soil (2), and the ocean (3). These interactions, moreover, are ancient: bacteriophages and bacteria have coevolved for more than 3 billion years (4, 5). Thus, despite their antagonistic relationship, the molecular mechanisms that govern the population dynamics of lytic bacteriophage and bacteria have evolved to stabilize their coexistence in the environment.

How is coexistence achieved? One standard view is centered on spatiotemporal evolutionary dynamics (6). For example, lytic bacteriophage can select for resistant bacterial mutants, which can subsequently lose resistance in bacteriophage-free environments, thereby maintaining populations of susceptible and resistant bacteria across environments (7, 8). Genetic resistance may rely on the mutation of the bacteriophage receptor (9), constitutive overproduction of extracellular polysaccharide (6) (which blocks the bacteriophage from its receptor), acquisition of restriction modification systems (10), or of CRISPR spacers (11). Ecological interactions, moreover, may influence these evolutionary dynamics. For example, because antibiotics (which are naturally produced by competing bacteria) and bacteriophages often interact with the same cell-envelope components, the evolution of antibiotic resistance can cause cross-resistance to bacteriophage (12).

The outcome of bacteriophage-bacteria competition is also strongly dependent on the environment. For example, in theory, bacterial growth on solid media could provide a mechanism of coexistence in the absence of genetic resistance if rapidly growing cells on the periphery of a bacterial microcolony shield interior cells (13). More importantly, bacteria can survive in the presence of bacteriophage when they adopt a slow-growing, sessile lifestyle within biofilms, which limits diffusion of the bacteriophage (14) and sterically blocks bacteriophage adsorption (15). Biofilms may also promote the type of evolutionary dynamics, described above, that lead to bacteria-bacteriophage coexistence (16). Biofilm synthesis can be induced by a variety of environmental stimuli (*e.g.,* starvation and quorum sensing), although not by bacteriophage itself. In other words, biofilms are an effective but not a specific defense against bacteriophage.

Along these lines, *E. coli* cells gain partial protection from bacteriophage when they coat themselves with colanic acid (“capsule”), which also aids in interspecies competition (17–19) and pathogenesis (20). Capsule is synthesized in response to cell-envelope damage via the Rcs phosphorelay pathway. As a result, capsule synthesis in response to sub-lethal envelope stress could, in principle, provide cross-protection against bacteriophage (21).

There are several key questions that remain unanswered with respect to bacteria-bacteriophage coexistence. First, each of the mechanisms by which bacteria cope with bacteriophage described above requires either bacteriophage resistance or a reduction of metabolic activity on the part of the bacterium. In either case, it is unclear how bacteriophages avoid extinction since they have relatively short lifetimes in the environment if they are not actively proliferating (22). Second, lytic bacteriophage and rapidly growing bacteriophage-susceptible bacteria are often found in abundance in the same niche. For example, high titers of one lytic bacteriophage active against *Vibrio cholerae* were measured in 100% of cholera patient stool samples, in which the number of susceptible bacteria was also very large (23). This outcome is not consistent with existing models of evolutionary predator-prey dynamics unless the rates of evolution are unrealistically high (24). Third, the presence of lytic bacteriophage can lead to a counterintuitive increase in the load of *V. cholerae* in a mouse model of cholera (25). Collectively, these observations point to unknown ecological interactions between the two microorganisms that allow them to proliferate in close proximity.

Here, we demonstrate that growth in confined, two-dimensional environments promotes “persistent” growth of *E. coli* in the presence of high titers of bacteriophage T7. We discovered that persistence occurs because physical confinement activates the Rcs phosphorelay pathway, leading to capsule synthesis, which reduces the rate of bacteriophage adsorption. Reduced adsorption, in turn, reduces the rate of bacterial lysis and causes bacterial proliferation to balance cell lysis such that susceptible bacteria within bacteriophage-infected microcolonies can grow rapidly and indefinitely. Bacteriophage persistence, therefore, is a novel innate mechanism by which bacteria can cope with lytic bacteriophage and which has important implications for our understanding of bacteriophage-bacteria ecology in real-world environments.

## RESULTS

### Confined bacteria grow persistently during bacteriophage predation

**Fig 1:**
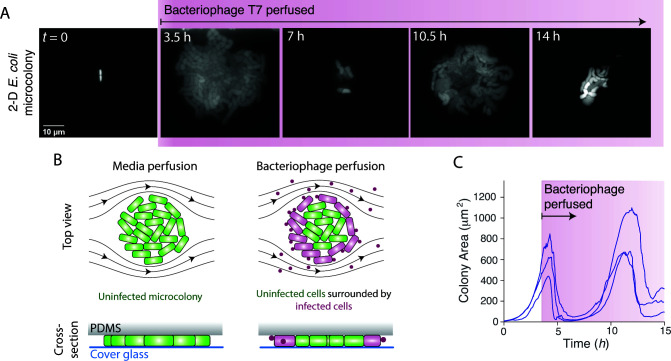
*E. coli* under confinement grows persistently during perfusion of bacteriophage T7. (**A**) Montage of a two-dimensional *E. coli* microcolony during perfusion of bacteriophage T7. (**B**) Schematic of the experimental design. (**C**) Colony area versus time for three *E. coli* microcolonies that grew under LB perfusion for 4 hours before being perfused with LB containing bacteriophage T7 indefinitely. Similar results were obtained for 15 experiments.

Bacteria-bacteriophage population dynamics have conventionally been quantified using bulk-culture assays (26). Bacteriophage predation of bacterial communities has been observed at the single-cell and single-virion level (27), but not with high temporal and spatial resolution simultaneously. Therefore, in order to examine the physiological basis for bacteria and bacteriophage coexistence, we used microfluidics and single-cell time-lapse microscopy to monitor the spatiotemporal dynamics of bacterial growth under bacteriophage predation for >10 hours continuously (Fig. 1A and B).

We first measured the dynamics of *E. coli* lysis by bacteriophage T7. Wild-type *E. coli* MG1655 has no mechanism of resistance or immunity to this virus. We allowed bacterial cells to proliferate exponentially for defined periods of time during constant perfusion with rich media before perfusing them indefinitely with media containing high-titer bacteriophage (10^7^ mL^−1^). Within the microfluidic perfusion chamber, the cells are trapped between a cover glass and a layer of polydimethylsiloxane (PDMS), which causes single cells to grow into two-dimensional (monolayer) microcolonies during the initial phase of growth (Fig. 1A and B). The subsequent perfusion of bacteriophage caused colonies to rapidly decrease in size (Fig. 1C). Cell lysis began at the colony periphery and progressed toward the center (Movie S1), suggesting that peripheral bacterial cells were temporarily protecting central cells by binding and sequestering the bacteriophage (Fig. 1B).

Surprisingly, many microcolonies re-established exponential growth after the initial period of rapid lysis despite being continuously perfused with bacteriophage (Fig. 1A and C). This “persistent” growth occurred at a frequency (8.6 ± 8.3%; mean ± 1 SD, *n* = 10 experiments) ≈10^5^ times higher than would be expected from the natural selection of resistant mutants (9, 28). Indeed, these microcolonies were still susceptible to bacteriophage since they underwent repeated periods of lysis and growth (Fig. 1C; Fig. S1A). The colony growth rate during the periods of growth was comparable to that of uninfected colonies (Fig. 1C), indicating that the bacteria were temporarily immune to the bacteriophage during these periods. Interestingly, the temporal dynamics of microcolony size often exhibited an oscillatory-like pattern that was synchronized between spatially separated microcolonies (Fig. 1C), suggesting that these dynamics were due to a stereotypical physiological response by the bacterium. Persistence occurred after as little as 5 minutes of growth in the perfusion chamber (Fig. S1A), and we observed persistent growth for up to 15 hours, which was long as we could continually image the bacteria.

**Fig 2:**
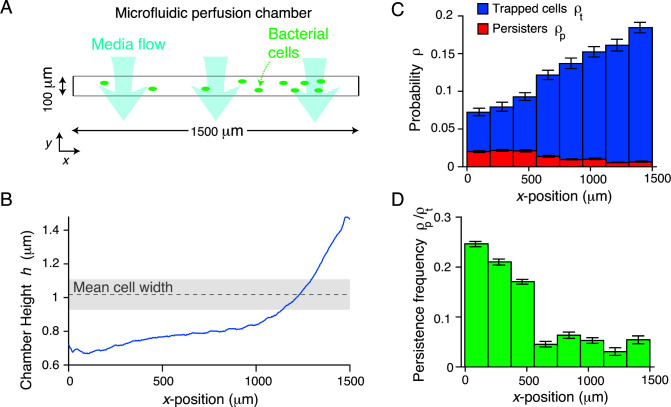
Persistent growth is correlated with the degree of mechanical confinement. (**A**) Diagram of the microfluidic perfusion chamber. The chamber is rectangular with a large aspect ratio, and the media is perfused parallel to the shorter (*y*) dimension. (**B**) Height of a perfusion chamber versus position along the longer (*x*) dimension of the chamber. (**C**) Mean spatial probability distribution of trapped cells (before bacteriophage perfusion) and persistent colonies (after bacteriophage perfusion) versus *x*-position in the perfusion chamber across six technical replicates. Error bars indicate ±1 SD. (**D**) The probability that a trapped cell would develop into a persistent microcolony versus position in the perfusion chamber. Error was propagated from data in **C**.

We confirmed that in bulk liquid culture, bacteriophage T7 rapidly kills our wild-type *E. coli* at a rate consistent with classic experiments (<10^−8^ resistant cells) (9). We therefore hypothesized that persistence specifically depended on confined growth. The rectangular microfluidic perfusion chambers we used are 1,500 × 100 µm (*x-y*, Fig. 2A) and are engineered with a height gradient (0.5 ≲ *h* ≲ 1.5) along their long axis (*x*-axis, Fig. 2B; Materials and Methods), which selectively traps bacterial cells in areas of the chamber where their width matches the chamber height. However, with lower probability cells become trapped in areas of the chamber with heights less than their width (Fig. 2A and C). The frequency at which this occurs depends on the flow rate used to load the cells into the chamber, allowing us to deliberately wedge cells into the low-height areas. Cellular growth rate did not depend on chamber height (Fig. S2A); however, prolonged periods of growth under high compression caused amorphous growth (Fig. 1A), consistent with previous studies (29). Interestingly, although cell density increased with chamber height, the probability for a cell to become persistent strongly decreased with height (Fig. 2C and D; Movie S2). That is, mechanical compression of the bacterial cell promoted persistent growth during bacteriophage predation. We observed a basal level of persistence, however, even for cells trapped in the tall (large-height) region of the chamber, indicating that surface sensing alone is sufficient to induce persistence.

### Rcs activation is required for compression-mediated persistence

**Fig 3:**
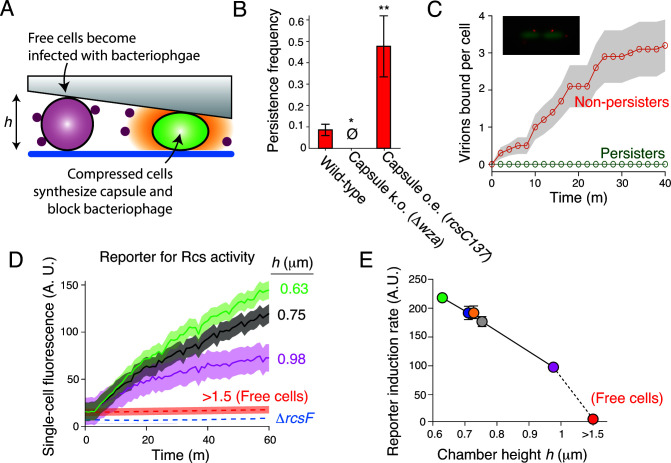
Mechanical compression induces Rcs activation. (**A**) Model for bacteriophage persistence. (**B**) Mean persistence frequency for wild-type *E. coli* and two isogenic mutants that do not express capsule (Δ*wza*) and overexpress capsule (*rcsC137*). Error bars indicate ±1 SEM across three technical replicates. ∅: no persistent colonies observed. ***: *P* < 0.001. (**C**) Population-averaged number of virions bound per cell versus time for cells that developed into persisters and cells that did not, during bacteriophage perfusion. Confidence intervals indicate ±1 SD across *n* = 10 cells. (Inset) Micrograph of two *E. coli* cells each with two bacteriophage T7 virions bound to them. (**D**) Population-averaged single-cell fluorescence versus time and perfusion chamber height for *E. coli* cells harboring a transcriptional reporter for Rcs activity. Confidence intervals indicate ±1 SD across *n* = 231,627,533,312,480,329 cells at each respective height. (**E**) Population-averaged single-cell induction rate of the reporter for Rcs activity versus perfusion chamber height, calculated from data shown in (D). Error bars indicate ±1 SD across *n* = 231,627,533,312,480,329 cells at each respective height.

We hypothesized that mechanical compression was inducing a transcriptional response that was protecting the bacteria from the bacteriophage and that the dynamics in colony size observed during persistence (Fig. 1C; Fig. S1A) reflected the dynamics of this transcriptional response. In a previous genome-wide screen, only a single gene, when overexpressed, provided resistance of *E. coli* to bacteriophage T7 (19). This gene, *rcsA*, encodes a transcription factor that, upon hetero-dimerization with a cognate transcription factor, RcsB, induces expression of an operon that synthesizes colanic acid (“capsule”), a polysaccharide that coats the cell surface (30). Furthermore, mutations that lead to constitutive overproduction of capsule result in bacteriophage resistance since capsule blocks the bacteriophage from binding to its receptors in the outer membrane (31). We therefore hypothesized that compression-mediated persistence was dependent on capsule synthesis (Fig. 3A). To test this, we measured the frequency of persistence in mutant bacteria that either lacked the ability to export capsule (*Δwza*) or possessed a constitutively active Rcs pathway (*rcsC137*), which increases the expression of RcsA-induced genes 30 times compared with wild-type bacteria (32). As hypothesized, capsule-less bacteria exhibited no persistence during bacteriophage predation, while constitutive Rcs activation led to a dramatic increase in the frequency of persistent microcolonies (Fig. 3B).

To confirm that capsule synthesis prevented bacteriophage adsorption, we perfused *E. coli* with fluorescently labeled bacteriophage (15) and measured adsorption dynamics in persistent and non-persistent cells (Fig. 3C). We found that bacteriophage rapidly adsorbed to non-persistent cells (0.08 ± 0.06 virions cell^−1^ minute^−1^; Fig. 3C and D), but did not adsorb to cells that developed into persistent microcolonies (Fig. 3C and D). Furthermore, bacteriophage adsorbed more slowly to non-persistent cells that constitutively synthesized capsules than to non-persistent wild-type cells (Fig. S2B). Together, these data directly demonstrate the requirement of capsule synthesis for bacterial persistence to bacteriophage.

### Mechanical compression causes signaling through the Rcs pathway

Our results led us to a model in which mechanical compression causes bacteriophage persistence by inducing capsule synthesis (Fig. 3A). Expression of capsule biosynthetic enzymes is induced by signaling through the Rcs phosphorelay pathway (30). This complex pathway is triggered by cell-envelope stress, such as that caused by cationic antimicrobial compounds (33), and ultimately induces up to ≈20 genes (including many not required for capsule synthesis), depending on the specific type of stress (33, 34).

To test whether mechanical compression activates Rcs signaling, we constructed a plasmid-based reporter in which the expression of super-folder GFP is controlled by the promoter of *rprA*, a gene whose transcription is induced by RcsB (35). We then measured reporter expression for cells under varying degrees of compression. We found that reporter expression was strongly negatively correlated with chamber height (Fig. 3D and E). Furthermore, reporter expression of cells trapped in the tallest area of the perfusion chamber was ≈50% of that of cells trapped in the shortest area of the cell, while reporter activity in bulk liquid culture was negligible (Fig. 3D), demonstrating that growth on surfaces alone is sufficient to elicit Rcs signaling. Mechanical compression did not activate Rcs signaling in a mutant bacterium lacking RcsF, the lipoprotein that initiates signaling in response to outer membrane damage (Fig. 3D), demonstrating that compression mediates capsule synthesis via the canonical mechanism.

**Fig 4:**
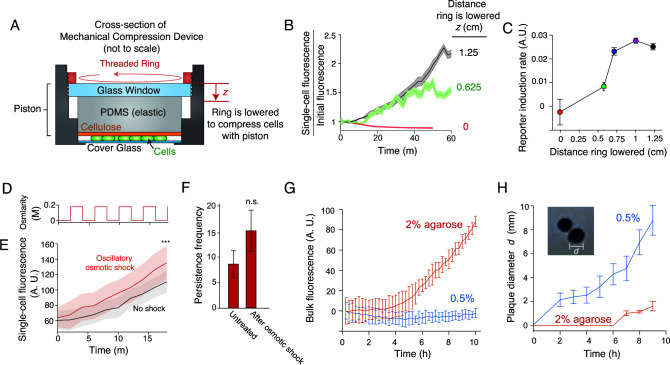
Several forms of mechanical stimulation cause persistence. (**A**) A novel mechanical compression device in which cells are compressed with an elastic piston by sandwiching the piston between a cover glass and a threaded aluminum ring that can be lowered (by screwing it into the cover glass holder) by defined amounts. (**B**) Population-averaged single-cell fluorescence versus time and degree of compression in the device shown in (A), for *E. coli* cells harboring a plasmid-based transcriptional reporter for Rcs activity. Confidence intervals indicate ±1 SD across cells at each respective height. (**C**) Population-averaged single-cell induction rate of the reporter for Rcs activity versus degree of compression. *n* = 10, 181, 459, 790, 534 cells at each respective compression level. Error bars indicate ±1 SEM. (**D**) Medium osmolarity versus time during a 200-mM oscillatory osmotic shock with a 4-minute period. (**E**) Population-averaged single-cell fluorescence versus time for untreated (non-shocked) *E. coli* cells harboring a plasmid-based transcriptional reporter for Rcs activity and for the same strain undergoing an oscillatory osmotic shock as shown in (D). Confidence intervals indicate ±1 SD across *n* = 35 and 15 cells for the two conditions, respectively. ***: *P* < 0.0001. (**F**) Mean persistence frequency for untreated (non-shocked) *E. coli* and for the same strain after having undergone an oscillatory osmotic shock as shown in (D). Error bars indicate ±1 SEM. (**G**) Mean bulk fluorescence versus time of *E. coli* cells harboring a plasmid-based transcriptional reporter for Rcs activity that are suspended in solid media containing different concentrations of agarose. Error bars indicate ±1 SD across three technical replicates. (**H**) Mean plaque size versus time when a single bacteriophage solution was diluted into suspensions of bacteria in solid media containing 2% and 0.5% agarose. Error bars indicate ±1 SD across three technical replicates. (Inset) Micrograph of two bacteriophage plaques.

To control for the possibility that compression-induced Rcs signaling was specific to the inorganic PDMS substrate used to trap cells, we invented a novel mechanical device that compresses cells with an organic, biocompatible substrate (Fig. 4A). In this device, cells are trapped between a cover glass and an elastic piston lined with cellulose. The piston can be controllably lowered to compress cells with defined (but uncalibrated) forces. As in the PDMS microfluidic device, the activation of our reporter was quantitatively dependent on the distance the piston was lowered (Fig. 4B and C), demonstrating that Rcs signaling is a generic result of mechanical compression.

### Multiple types of mechanical stimulation can elicit Rcs signaling and induce persistence

Surface sensing and mechanical compression are two of many examples of mechanical stimuli that bacteria may sense in their environment. Thus, we next questioned whether other types of mechanical perturbation also induce Rcs signaling and bacteriophage persistence. We first tested the effect of osmotic fluctuations, an environmental perturbation that causes variation in the intracellular turgor pressure within cells (36). To do so, we subjected cells to an oscillatory osmotic shock (37) (Fig. 4D) immediately before bacteriophage perfusion. This treatment caused modest increases in both the rate of Rcs activation and the frequency of persistence (Fig. 4E and F).

We next questioned whether persistence at the single-cell level could affect large-scale bacterial population dynamics or whether it was specific to microscale colonies. To do so, we used a bulk-culture method (38) to assay the effects of mechanical stimulation on Rcs activation and bacterial proliferation during bacteriophage predation. First, we suspended cells in agarose gels of varying stiffnesses and assayed Rcs activation in our reporter strain using a fluorescence plate reader. Similar to the case of mechanical compression (Fig. 3D and E), the rate of induction was much higher when cells were grown in stiff 2% agarose media than in soft 0.5% media (Fig. 4G). Although we could not directly quantify single-cell persistence from this bulk-culture assay, we quantified the rate of bacteriophage proliferation by measuring the growth rate of bacteriophage plaques on these solid-media bacterial suspensions. As predicted, the rate of plaque growth was much slower on bacterial suspensions in stiff media than in soft media (Fig. 4H). Together with the increase in persistence in response to oscillatory osmotic shock (Fig. 4D through F), this result demonstrates that various sources of mechanical forces can induce persistence.

## DISCUSSION

We discovered a new mechanism by which mechanically stimulated bacteria can grow persistently—and indefinitely—during lytic bacteriophage predation. They accomplish this by producing extracellular capsule in response to mechanosensation by the Rcs pathway, thereby slowing the dynamics of predation. In doing so, both the bacteria and the bacteriophage can proliferate simultaneously in the same environment.

We called this phenomenon “persistence” because it shares the essential attribute of antibiotic persistence (39): that bacteria can temporarily survive the insult without a specific mechanism of resistance. Just as toxin/antitoxin-mediated antibiotic persistence (40) is not a specific defense against antibiotics (but rather a mechanism for stochastic dormancy), capsule-mediated bacteriophage persistence is not a specific defense against bacteriophage but a response to mechanical touch that may serve other purposes as well. For example, the Rcs pathway promotes persistent *Salmonella* infections (41), which could rely on the mechanical sensitivity of this pathway in the gut. It is also possible that bacteriophage persistence is cross-protective against certain toxic compounds to which capsule is impermeable (42).

In one sense, capsule-mediated persistence is similar to the protection that bacteria gain from bacteriophage by living in biofilms. In both cases, environmental stimuli induce the synthesis of an extracellular factor that inhibits bacteriophage replication, and thus biofilms provide a form of persistence. However, the most remarkable feature of mechanically induced bacteriophage persistence, which distinguishes it from both biofilm-based bacteriophage protection and antibiotic persistence, is that it allows exponentially growing bacteria to survive bacteriophage. On the one hand, this feature serves a clear function for non-resistant bacteria by providing niches (e.g., solid media or strongly fluctuating environments) where they can rapidly grow in spite of bacteriophage. On the other hand, this also allows bacteriophages to proliferate without extinguishing their host. Together, these characteristics of bacteriophage persistence promote stable coexistence of the two microbes.

Phylogenetically, the Rcs phosphorelay pathway is specific to enteric bacteria (41). Capsule, however, is much more widespread and therefore other taxa may grow persistently during bacteriophage predation using non-Rcs envelope stress response pathways. For example, the two-component system VxrAB, which senses envelope stress in *V. cholerae*, is also sensitive to mechanical compression, which could mediate persistence in this bacterial pathogen (43).

The molecular mechanism of mechanosensation by the Rcs pathway is an open question. Since mechanical signaling requires RcsF, it is likely that compression causes outer membrane damage that resembles damage caused by cationic antimicrobial compounds such as colistin. Alternatively, it was recently demonstrated that bacterial mutants with increased cell width induce Rcs signaling because they have a thinner periplasm, which brings RcsF closer to its phosphorelay target IgaA (44). This study independently discovered that mechanical compression induces Rcs signaling and attributed this dependence to the increased (lateral) width of compressed cells. While periplasmic thinning and outer membrane damage are not mutually exclusive—and therefore both could underlie compression-mediated Rcs signaling—our observation that surface sensing alone rapidly induces Rcs signaling (Fig. 3D) suggests that cell widening is not necessary for this transduction during compression.

In contrast to colistin, mechanical stimulation does not cause a strong reduction in cellular growth rate, and the Rcs pathway responds to even minor mechanical stimuli (growing on a surface, Fig. 3D and E). This suggests that the Rcs pathway acts as a sensitive, adaptive form of mechanosensation in addition to an emergency stress response. If so, this would add to a growing body of evidence that bacteria are exquisitely sensitive to their mechanical environment and execute sophisticated transcriptional programs upon mechanosensation (45). For example, *Pseudomonas aeruginosa* executes a virulence program when it senses surfaces (46). Since in certain cases capsule is a virulence factor (47), our findings suggest that mechanical compression may also contribute to bacterial pathogenesis. Indeed, since bacteriophages are often present during bacterial pathogenesis (48), protection from them may be required for virulence in certain cases. On this note, our finding has clear implications for the prospects of phage therapy: the ability of the bacteriophage to overcome capsule must now be considered.

Interestingly, the variation in the induction of our RcsB-induced reporter was relatively low for a given degree of mechanical compression (Fig. 3D), and yet only a relatively small fraction of cells became persistent, regardless of compression level (Fig. 2D). This suggests that there is a stochastic component to capsule synthesis downstream of RcsB that influences persistence statistics. We find it likely that this discrepancy is due to RcsA dynamics, since this protein also influences capsule synthesis. If so, it will be interesting to use our mechanical assays to interrogate the mechanism of information processing that *E. coli* uses to filter RcsA and RcsB dynamics in order to trigger capsule synthesis.

Bacteriophages are present in most natural environments where bacteria thrive (49): in the gut, the soil, and virtually all bodies of water. In most of these environments, bacteria are directly subjected to mechanical forces either by other cells, by their environment, or by osmotic fluctuations. We propose that mechanically induced bacteriophage persistence is a previously unappreciated phenomenon that underlies the coexistence of bacteria and bacteriophage in many of these environments.

## MATERIALS AND METHODS

### Bacterial strains and culture conditions

All strains used in this study are listed in Table S1. All experiments were conducted with *E. coli* MG1655 or isogenic mutants. Bacteria were cultured in lysogeny broth (LB) with appropriate antibiotic selection at 37°C with shaking at 180 RPM, unless otherwise noted. Capsule-less cells were the same size as wild-type cells demonstrating that they did not experience less compression than wild-type cells (Fig. S2C and D).

### Bacteriophage propagation and purification

Bacteriophage T7 was propagated using standard techniques. Autoclaved molten LB top agar (0.5% agarose) was brought to 65°C in a water bath. Serial 10× dilutions of bacteriophage T7 suspensions were made in bacteriophage buffer (10-mM TRIS at pH 7.5, 10-mM MgSO_4_, 70-mM NaCl). In addition, 10 µL of each bacteriophage dilution was added to 200 µL of overnight *E. coli* MG1655 culture and incubated at room temperature for 20 minutes, and 5 mL of molten top agar was added to each culture and poured over a pre-warmed LB plate. Plates were incubated overnight at 37°C. The following day, 5 mL of bacteriophage buffer was added to the top of the plate that had the highest number of individual plaques that could still be counted and was incubated at room temperature for 2 hours. The phage buffer was then removed with a syringe and sterilized through a 0.2-µm syringe filter.

### Strain and plasmid construction

All plasmids used in this study are listed in Table S2. Strain ER396 (MG1655, Δ*wza::Kan*) was generated by P1 transduction between ER373 (BW25113, Δ*wza::Kan*) and MG1655. Strain ER473 (MG1655, Δ*rcsF:Kan*) was generated by P1 transduction between ER367 (BW25113, Δ*rcsF::Kan*) and MG1655.

Plasmid pGM04 was generated by Gibson assembly (50) of PCR amplicons of the backbone of pZS21 (51), the promoter of of *rprA* (60-base pair upstream of the *rprA* start codon), the ribosomal binding site used in reference 35, and monomeric super-folder GFP (msfGFP). The *rprA* promoter was amplified from *E. coli* MG1655, and msfGFP was amplified from NO34 (47).

### Persistence assay

Prior to the experiment, 1 mL of LB was inoculated with 10 µL of overnight *E. coli* culture and incubated at 37°C with shaking for 2 hours. The cell inlet well of a CellASIC bacterial microfluidic plate (B04A) was loaded with 200-µL LB; two media perfusion wells were loaded with 200-µL of LB and LB containing a high-titer (≥10^7^ mL^−1^) of bacteriophage T7. The plate was then pre-warmed at 37°C in the microscope incubation chamber for 45 minutes, after which 2 µL of the exponentially growing bacterial culture was added to the loading well (1:100 dilution). The perfusion wells were then primed for 30 minutes in the microscope incubation chamber. The ONIX microfluidic perfusion system was then used to load cells into the perfusion chamber at high pressure (10 psi) to drive cells into areas of the plate that had low chamber heights. Trapped cells were perfused with LB for defined periods of time, followed by indefinite perfusion of LB-containing bacteriophage.

Bacterial cell growth and reporter fluorescence were recorded using a Nikon Ti2 inverted microscope and a Photometrics sCMOS camera. Fluorescence was excited using Aura Light Engine LED light source (Lumencore).

To subject cells to oscillatory osmotic shock prior to measuring persistence, LB-containing 200-mM sorbitol was also loaded into a third media perfusion well on the microfluidic plate. After loading cells into the perfusion chamber, they were subjected to 10 cycles of a 200-mM oscillatory osmotic shock with 4-minute period before LB-containing bacteriophage was perfused indefinitely (with no osmotic shock).

### Perfusion chamber height determination

To determine perfusion chamber height before a persistence experiment, a fluorescent tracer dye (Alexa Fluor 647 succinimidyl ester) was perfused into the chamber and a single stitched epifluorescence micrograph was taken of the entire chamber at ×100 magnification. The intensity of the tracer dye as a function of *x*-position across a single slice (*y*-position) of the perfusion chamber was computationally measured from this image. To calibrate the intensity profile with the chamber height, upon loading cells into the chamber during the subsequent experiment, we found the largest *x*-position (corresponding to the tallest chamber height) at which cells became trapped and calibrated that height with the diameter of the cells, which we measured from the micrographs.

### Mechanical compression device

The frame for the mechanical compression device was machined from aluminum and anodized. The specifications for the frame are shown in Fig. S3. The device holds a 25-mm diameter round cover glass. The piston consists of a 30-mm diameter circular PDMS plug, which was lined with dialysis membrane by plasma bonding the two together, and a circular 1.5-in diameter glass window. In addition, 10 µL of exponentially growing *E. coli* cells were placed onto the cover glass. The cellulose-PDMS plug was placed on top of the cells, and the glass window was placed on top of the plug. The aluminum cover glass holder is threaded, and a threaded aluminum ring was screwed into it, thereby compressing the piston by defined amounts.

### Fluorescent labeling of bacteriophage

Covalent fluorescent labeling of the capsid of bacteriophage T7 was performed as described by Vidakovic et al. (15). Briefly, 100 µL of a purified bacteriophage (10^11^ pfu mL^−1^) mixed with sodium bicarbonate (0.1 M final) was incubated with 0.1-mg Alexa Fluor 647 succinimidyl ester for 1 hour at room temperature under continuous shaking and protected from light. The reaction mixture was dialyzed against PBS to separate bacteriophages from unbound dye for 2 days using 20,000 molecular weight cutoff (MWCO) Slide-A-Lyzer cassettes. Fluorescently labeled bacteriophages were stored at 4°C. Bacteriophage binding was counted manually.

### Computational image analysis

Tracking of the size of persistent microcolonies and quantification of single-cell fluorescence in the Rcs reporter was performed using custom MATLAB scripts.

### Bulk culture measurements of Rcs reporter fluorescence and plaque formation

To measure the induction of Rcs signaling in the reporter strain ER450 growing in solid media of varying stiffness, 200-µL molten LB media containing 0.5% or 2% agarose was equilibrated to 50°C and mixed with 10 µL of overnight bacterial culture in a 96-well plate. Fluorescence measurements were taken with a Tecan Spark Fluorescence plate reader at 15-minute intervals.

To measure plaque formation in agar of varying stiffness, LB agar plates were pre-warmed to 37°C, and molten LB media containing 0.5% or 2% agarose was equilibrated to 50°C. In addition, 200 µL of overnight bacterial culture was mixed with 20 µL of bacteriophage T7, serially diluted to 1,000 pfu/mL, and incubated with the bacteria at room temperature for 20 minutes. Moreover, 5 mL of molten agarose media was added to the culture tube, and the mixture was poured over the pre-warmed agarose plates. Photos of the plaques were taken with a digital camera every hour for 9 hours.

## Data Availability

Data and plasmids from this study can be obtained upon request to the corresponding author. MATLAB code used for data analysis is available at https://github.com/TheRojasLab/BacterialCompression.

## SUPPLEMENTAL MATERIAL

Supplemental material mbio.02766-22-s0001.docx Fig. S1 to S3, Tables S1 and S2, and legends for Movies S1 and S2.

Movie S1 mbio.02766-22-s0002.avi Time-lapse micrograph (100X magnification) of *E. coli* cells expressing cytosolic GFP growing in a microfluidic perfusion chamber for 4 hours before being perfused with bacteriophage (not visible) for 11 hours.

Movie S2 mbio.02766-22-s0003.avi Time-lapse micrograph (10X magnification) of *E. coli* cells expressing cytosolic GFP growing in a microfluidic perfusion chamber for 5 minutes before being perfused with bacteriophage (not visible) for 3 hours.

ASM does not own the copyrights to Supplemental Material that may be linked to, or accessed through, an article. The authors have granted ASM a non-exclusive, world-wide license to publish the Supplemental Material files. Please contact the corresponding author directly for reuse.
