# Supplementary material for: Mechanosensation induces persistent bacterial growth during bacteriophage predation
Source: mBio. 2023 Nov 1;14(6):e02766-22. doi: 10.1128/mbio.02766-22 (PMC10746221; doi:10.1128/mbio.02766-22)
Supplement: Supplemental material — Fig. S1 to S3, Tables S1 and S2, and legends for Movies S1 and S2. [file mbio.02766-22-s0001.docx]

**Supporting Information for:**

Mechanical compression induces persistent bacterial growth during bacteriophage predation

Guy Mason^1^, Matthew J. Footer^2^, and Enrique R. Rojas^1,^*****

^1^Center for Genomics & Systems Biology, Department of Biology, New York University, 12 Waverly Place, New York, NY 10003, USA

^2^Department of Biology and Howard Hughes Medical Institute, University of Washington, Seattle, WA, USA

*Email: rojas@nyu.edu

**This PDF file includes:**

Figures S1 and S2

Tables S1 and S2

Legends for Movies S1 to S2

SI References

**Other supporting materials for this manuscript include the following:**

Movies S1 and S2

Fig. S1. Persistence can occur after as little as 5 minutes of growth in the perfusion chamber. Colony area versus time for three *E. coli* microcolonies that grew under LB perfusion for 5 minutes before being perfused with LB containing bacteriophage T7 indefinitely.

Fig. S2. Capsule expression slows the rate of bacteriophage binding in non-persistent cells. A) Elongation rate versus *x-*position in the microfluidic device, which is correlated with chamber height. Error bars indicate ±1 s.d.  *n* = 89, 229, 498,265, 637, 813, 478 cells at each respective height. B) Population-averaged number of virions bound per cell versus time for non-persistent wild-type cells and non-persistent mutant bacteria that constitutively overexpressed capsule (*rcsC137*). Confidence intervals indicate ±1 s.d. across *n* = 10 cell for each strain. C) Population-averaged length for wild-type cells and cells that do not export capsule (*Δwza*). Confidence intervals indicate ±1 s.d. *n* = 85 and 132 cells for wild type *Δwza*, respectively. C) Population-averaged width for wild-type cells and cells that do not export capsule (*Δwza*). Confidence intervals indicate ±1 s.d. *n* = 85 and 132 cells for wild type *Δwza*, respectively.

**Fig. S3. Specifications for the frame of the mechanical compression device.**

Table S1. Bacterial strains used in this study.

| Strain | Species | Genotype | Source/Reference |
| --- | --- | --- | --- |
| MG1655 | *E. coli* | WT | Laboratory stock |
| ER373 | *E. coli* | BW25113, Δ*wza::Kan* | *E. coli* Genetic Stock Center |
| ER473 | *E. coli* | BW25113, Δ*rcsF::Kan* | *E. coli* Genetic Stock Center |
| ER202 | *E. coli* | MG1655, pGM01 | Laboratory construct |
| ER396 | *E. coli* | MG1655, Δ*wza::Kan* | Laboratory construct |
| ER473 | *E. coli* | MG1655, Δ*rcsF::Kan* | Laboratory construct |
| 22563 | *E. coli* | MG1655, *rcsC137::Cm* | Gift from the Gottesman lab^1^ |
| ER450 | *E. coli* | MG1655, pGM04 (pZS21-*prprA-msfgfp* | Laboratory construct |

Table S2. Plasmids used in this study

| Plasmid | Genotype | Source/Reference |
| --- | --- | --- |
| pZS21 | pZS21^2^ | Gift from the Silhavy Lab |
| pGM01 | pZS21::*gfp* | Laboratory construct |
| pGM04 | pZS21::*prprA-msfGFP* | Laboratory construct |

Movie S1 (separate file). Time-lapse micrograph (100X magnification) of *E. coli* cells expressing cytosolic GFP growing in a microfluidic perfusion chamber for 4 hours before being perfused with bacteriophage (not visible) for 11 hours.

Movie S2 (separate file). Time-lapse micrograph (10X magnification) of *E. coli* cells expressing cytosolic GFP growing in a microfluidic perfusion chamber for 5 minutes before being perfused with bacteriophage (not visible) for 3 hours. Persistent microcolonies are enriched on the left side of the image where the height of the perfusion chamber is smallest.

**SI References**

1. Majdalani, N., Heck, M., Stout, V. & Gottesman, S. Role of RcsF in Signaling to the Rcs Phosphorelay Pathway in *Escherichia coli*[.](http://paperpile.com/b/ZWQiXk/b8oc) *Journal of Bacteriology* vol. 187 6770–6778 (2005).
2. Lutz, R. & Bujard, H. Independent and tight regulation of transcriptional units in Escherichia coli via the LacR/O, the TetR/O and AraC/I1-I2 regulatory elements. *Nucleic Acids Res.* **25**, 1203–1210 (1997).
